# The brain can develop conflicting multisensory principles to guide behavior

**DOI:** 10.1093/cercor/bhae247

**Published:** 2024-06-15

**Authors:** Scott A Smyre, Naomi L Bean, Barry E Stein, Benjamin A Rowland

**Affiliations:** Department of Neurobiology and Anatomy, Wake Forest School of Medicine, Medical Center Blvd., Winston Salem, NC 27157, United States; Department of Neurobiology and Anatomy, Wake Forest School of Medicine, Medical Center Blvd., Winston Salem, NC 27157, United States; Department of Neurobiology and Anatomy, Wake Forest School of Medicine, Medical Center Blvd., Winston Salem, NC 27157, United States; Department of Neurobiology and Anatomy, Wake Forest School of Medicine, Medical Center Blvd., Winston Salem, NC 27157, United States

**Keywords:** cross-modal, integration, noise-rearing, vision, hearing

## Abstract

Midbrain multisensory neurons undergo a significant postnatal transition in how they process cross-modal (e.g. visual–auditory) signals. In early stages, signals derived from common events are processed competitively; however, at later stages they are processed cooperatively such that their salience is enhanced. This transition reflects adaptation to cross-modal configurations that are consistently experienced and become informative about which correspond to common events. Tested here was the assumption that overt behaviors follow a similar maturation. Cats were reared in omnidirectional sound thereby compromising the experience needed for this developmental process. Animals were then repeatedly exposed to different configurations of visual and auditory stimuli (e.g. spatiotemporally congruent or spatially disparate) that varied on each side of space and their behavior was assessed using a detection/localization task. Animals showed enhanced performance to stimuli consistent with the experience provided: congruent stimuli elicited enhanced behaviors where spatially congruent cross-modal experience was provided, and spatially disparate stimuli elicited enhanced behaviors where spatially disparate cross-modal experience was provided. Cross-modal configurations not consistent with experience did not enhance responses. The presumptive benefit of such flexibility in the multisensory developmental process is to sensitize neural circuits (and the behaviors they control) to the features of the environment in which they will function. These experiments reveal that these processes have a high degree of flexibility, such that two (conflicting) multisensory principles can be implemented by cross-modal experience on opposite sides of space even within the same animal.

## Introduction

The adult brain integrates information across its multiple senses in a variety of functional contexts to enhance perception and behavior beyond that which can be accomplished with a single sense (Stein and Meredith 1993; Stein 2012). This capability for “multisensory enhancement” is believed to reflect the synergistic processing of cross-modal (e.g. visual and auditory) sensory signals that are inferred to be mutually informative about a common event (Körding et al. 2007; Stein et al. 2014; Kayser and Shams 2015; Rohe and Noppeney 2015; Cao et al. 2019).

The requisite multisensory inference is not an inherent property of the brain and, in studies with an animal model, has been shown to develop based on early experience with cross-modal stimuli (Wallace and Stein 1997, 2000, 2001, 2007; Wallace et al. 2006; Xu et al. 2012). Congenital conditions that compromise early visual–auditory experience (e.g. deafness or blindness) in humans can also lead to persistent impairments in integrating information from these modalities long after the unisensory defects are ameliorated (Putzar et al. 2007, 2010; Nava et al. 2014; Collignon et al. 2015; Stevenson et al. 2017; Senna et al. 2021). Similarly, animals raised in conditions that compromise visual–auditory experience (e.g. darkness or omnidirectional masking sound) are unable to use visual–auditory information synergistically to enhance behavioral performance (Smyre et al. 2021; Bean et al. 2022). The inability to benefit from the presence of cross-modal cues in the absence of appropriate prior cross-modal experience appears to be common across mammals, and perhaps all species capable of multisensory integration (Stein et al. 2014; Bruns and Röder 2023).

This perceptual/behavioral “dysfunction” is paralleled by anomalies in midbrain multisensory neural circuits. For example, in normally developed superior colliculus (SC) neurons that support detection and orientation behaviors, spatiotemporally congruent (i.e. mutually informative) visual–auditory stimuli elicit responses that are more robust than either stimulus component individually (Meredith and Stein 1983, 1986a; Wallace et al. 1998; Jiang et al. 2001, 2002; Burnett et al. 2004, 2007; Rowland and Stein 2007; Rowland et al. 2007a; Stein et al. 2014; Miller et al. 2017). However, in the absence of visual–auditory experience, multisensory SC neurons process visual–auditory inputs as competitive or mutually inhibitory (Yu et al. 2019; Wang et al. 2020; Smyre et al. 2021; Bean et al. 2022). This competitive computation persists even when animals are placed in a normal environment as adults (Xu et al. 2017). Although this neural defect appears to be durable, exposing the animals to simple flashes and broadband noise bursts that are congruent in space and time can reverse it, and lead to the development of SC multisensory enhancement capabilities in both dark-reared and noise-reared animals (Yu et al. 2010; Xu et al. 2017). There is evidence that extensive (years) of experience in an normal environment might also promote the development of enhanced multisensory perception and behavior in compromised adults (Nava et al. 2014; Rowland et al. 2014; Senna et al. 2021).

How does the brain use cross-modal experience to develop multisensory integration capabilities? One perspective is that repeated exposure to cross-modal stimuli in reliable configurations provides sufficiently strong evidence to instantiate a general assumption that the senses are mutually informative. This assumption could then be used to synthesize information in novel circumstances, allowing even limited experience to create a broad capability for multisensory enhancement. Another perspective is that the rules developed from cross-modal experience is limited to the specific features of the exposure stimuli (e.g. specific shape-frequency pairings. However, this would require far more experience to develop this broad capability and would also depend on the relative statistics of different cross-modal experiences. Nevertheless, the final configuration would better reflect the interaction of each individual with its particular environment. Given the complex nature of cross-modal experience received during normal development, it is challenging to assess these possibilities in humans or animals reared in normal environments.

To examine these possibilities experimentally, cats were reared to adulthood in omnidirectional sound. This precludes the development of visual–auditory multisensory integration capabilities that enhance SC physiological responses and SC-guided detection/localization behaviors (Xu et al. 2014, 2017; Bean et al. 2022). These “naïve” animals were then provided with specific unisensory and cross-modal experiences that would lead to different maturational endpoints if one or the other of these developmental perspectives were correct.

## Methods

All procedures were conducted in accordance with the Guide for the Care and Use of Laboratory Animals (National Institutes of Health Publication) and an approved Institutional Animal Care and Use Committee protocol at Wake Forest University School of Medicine, an Association for Assessment and Accreditation of Laboratory Animal Care-accredited institution. Six mongrel cats (three males, three female) were used in these studies. They were motivated by food rewards (2–3 × 175 mg pellets, Science Diet) and were maintained within 80% of their baseline body weight.

###  

#### Experimental summary

Noise-reared animals’ ability to detect and localize auditory, visual, and different configurations of visual–auditory pairings were evaluated before, during, and after they received repeated, high-density sensory experience at a single position in the left and right hemifields. The structure of this experience differed on each side of space depending on the experimental objective. The objective of experiment 1 was to contrast the effects of spatially congruent cross-modal experience with unisensory experience, and animals were trained to localize both lights and sounds (“redundant targets”). The objective of experiment 2 was to contrast the effects of spatially congruent and spatially disparate cross-modal experience. These animals were trained to localize sounds (“auditory targets”) but not lights, so that when spatially disparate visual–auditory configurations were tested the correct response was unambiguous.

#### Animals and rearing condition

Animals were raised to adulthood (~1.5 yr) in an omnidirectional sound room containing six speakers (Fountek RM-6 K) mounted at ceiling level on all four walls and connected to a broadband noise generator (20–20,000 Hz, Coulbourn Instruments S81–02) and amplifier (Pioneer Audio Multi-Channel Receiver 5x-316) to produce a constant broadband noise at ~83 dB at cage level. This sound was continuous (24 h a day/7 d a week) and sufficient to mask most patterned auditory stimuli (Chang and Merzenich 2003; Efrati and Gutfreund 2011; Xu et al. 2014, 2015; Bean et al. 2022). Animals remained in the rearing environment for the duration of the study except for periods of training, exposure, and testing. Three animals (two male, one female, NR1–3) participated in experiment 1 and three (one male, two females, NR4–6) participated in experiment 2.

#### Apparatuses

**Fig. 1:**
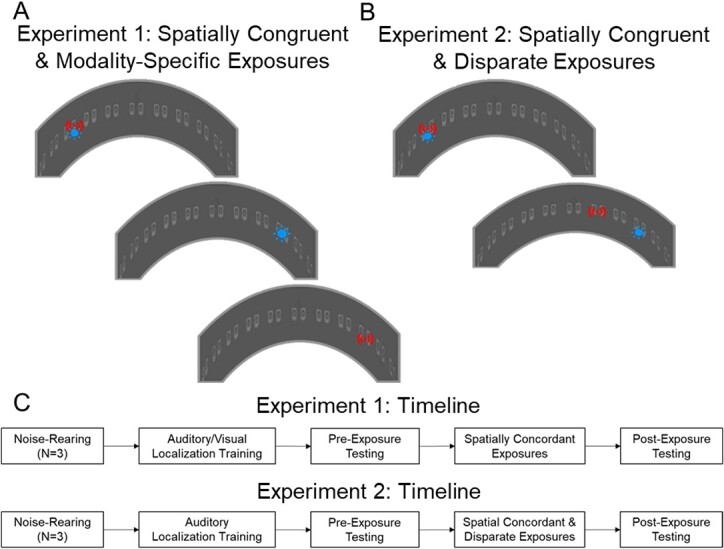
**Procedure sequences for experiments 1 and 2. A/B)** Detection/localization tasks in experiment 1 were performed in a perimetry apparatus with LEDs and speakers at locations spanning the central 120° of space in 15° intervals. Exemplar LEDs are indicated with blue icons, exemplar speakers with red icons. **A)** For each animal in experiment 1, one hemifield was designated “multisensory” and the other “unisensory.” the former was used to provide exposure to the visual–auditory stimulus combination and the latter to its component cues individually. These cross-modal and modality-specific trials were interleaved and the multisensory and unisensory hemifields varied across animals. **B)** In experiment 2 both hemifields were designated “multisensory.” one was used to present cross-modal stimuli in spatiotemporal congruence-45° from midline, and the other to present them in temporal congruence but spatial disparity (the auditory stimulus at +15° and the visual at +45°). Animals were required to approach the auditory cue when presented and remain at the start position (No-Go response) on catch trials to receive a food reward. **C)** Shown is the rearing, training, exposure, and testing sequence.

Behavioral training and testing were conducted using two perimetry apparatuses containing complexes of LEDs and speakers mounted at eye level (Fig. 1). Experiment 1 used a 110.6 cm diameter perimetry with complexes that were spaced at 15° intervals spanning the central 180° and consisted of a 9 cm × 6 cm assembly of two speakers (Panasonic model 4D02C0) and three LEDs (Lumex Opto/Components; model 67–1102-ND) (Gingras et al. 2009; Rowland et al. 2014; Dakos et al. 2020; Bean et al. 2021, 2022; Smyre et al. 2021). Only the left-most speaker and LED in a complex were used in the study. The apparatus for experiment 2 was slightly smaller (104.6 cm diameter) with complexes of only a single LED (Lumex Opto/Components; model 67–1102-ND) and speaker (Gikfun 2” 4Ohm 3 W-EK1726) mounted at 15° intervals.

#### Initial training

Animals were transported to the exposure apparatus each weekday in shrouded carriers. Transports contained “white” masking noise (Noise Gen App on Apple iPhone 11, 83.1–84.1 dB). The animals were trained in the light with “background” noise provided by a noise generator (Noise Gen App on Apple iPhone SE) connected to two speakers (Harman Kardon model HK206) mounted on top of the perimetry apparatus (see Fig. 1C). All animals were initially trained to fixate on 0° before being trained in one of two established detection/localization paradigms. Animals in experiment 1 were trained in a “redundant targets” paradigm. They were first trained to approach a highly visible (“full” intensity) visual target (50 ms LED flashes, 2.0–3.7 cd/m^2^) presented at a randomly selected location between −60° (left) and + 60° (right), excluding 0°, then trained in similar fashion to approach highly audible (“full” intensity) auditory targets (50 ms broadband noise burst, ~61 dB SPL). Animals in experiment 2 were trained in an “auditory targets” paradigm. They were trained to localize full intensity auditory stimuli (100 ms broadband noise burst, ~61 dB SPL) between −45° (left) at +45° (right), excluding 0°, but were never presented with a visual training stimulus. All training trials for all animals were interleaved with catch trials (no stimulus) in which the animal had to remain at the start position (“No-Go”) to receive a food reward. Trials were initiated by the experimenter depressing a foot pedal. All animals were trained until satiated each day and then transported in the same carriers back to their home environment. Once animals reached criterion performance (>80% correct responses) they proceeded to Pre-Exposure Testing.

#### Preexposure testing

The intensities of target stimuli (experiment 1: visual and auditory, experiment 2: only auditory) were reduced to those eliciting correct responses on 30–45% of trials (in a block of 20) for each animal/location. Background noise was at ~84 dB when determining baseline visual intensities and ~56 dB when identifying the auditory baseline intensities (to avoid masking them). Combinations of these “low” intensity stimuli maximized the opportunity of revealing the capability for multisensory enhancement (Meredith and Stein 1986a; Stein et al. 1989).

In the testing block, stimuli tested at multiple locations (experiment 1: between −60° and 60°, experiment 2: between −45° and 45°) were auditory stimuli at low intensity, visual stimuli at low (experiment 1) or full (experiment 2) intensity, a spatially congruent and synchronous visual–auditory combination (both experiments), or a spatially disparate (30° separation) and synchronous cross-modal combination (only experiment 2). Stimulus-containing trials were randomly interleaved with occasional catch trials (~8–12% incidence). Individual auditory (A) and visual (V) stimuli at respective locations X° and Y° are denoted as AX°, VY°, while a cross-modal (synchronous) presentation including these same stimuli is denoted as AX°/VY°.

Animals were rewarded for correctly approaching target stimuli and for remaining at the start position (No-Go responses) on catch trials. Animals in experiment 2 (auditory targets) were not rewarded for approaching a visual stimulus. A minimum of 48 trials were conducted for each location/condition/animal. Initial training and baseline testing data for the animals in experiment 1 have been previously described (Bean et al. 2022). Animals then began the next phase of experimentation (Cross-modal Exposure).

#### Cross-modal exposure

Animals were provided with blocks of exposures to visual, auditory, or visual–auditory stimulus combinations. All trial types were interleaved with each other and catch trials. Background sound intensity was always ~56 dB during exposures and all stimuli were presented at full intensity (visual = 2.0–3.7 cd/m^2^; auditory = ~61 dB SPL). The specific stimulus configurations in the exposure block were different in the two experiments and, within an experiment, were different on the two sides of space (− denotes the left hemifield and + denotes the right). These configurations are depicted graphically in Fig. 1.

To compare the effects of cross-modal vs. modality-specific exposures, animals in experiment 1 received exposures to a spatially congruent visual–auditory stimulus (synchronous, 50 ms duration) at a single location in one hemifield (+45° for NR1 and NR2; −45° for NR3) and the component visual and auditory stimuli presented individually at the homotopic location in the opposite hemisphere (−45° for NR1 and NR2; +45° for NR3). The actual sides receiving each type of experience differed across animals, but, because results did not differ by hemifield, the side of space receiving congruent cross-modal exposure is identified below as “left.” To compare the effects of spatially congruent vs. disparate cross-modal exposures, animals in experiment 2 received exposures to a spatially congruent visual–auditory stimulus (synchronous, 100 ms duration) at a single location in one hemifield (−45°) and a synchronous (100 ms) but spatially disparate configuration in the opposite hemifield (the auditory stimulus was at +15° and the visual at +45°, A15°/V45°).

A total of 60 exposures per hemifield and 12 catch trials were presented each day. As in preexposure testing, correct approaches to target stimuli or No-Go responses on catch trials received a food reward. The development of multisensory integration capabilities (i.e. enhanced multisensory performance) was tracked in experiment 1 by interleaving 2 daily sessions of a battery of low intensity visual, auditory, visual–auditory, and catch trials with each block of 2–4 exposure sessions.

#### Postexposure testing

After the cross-modal exposure block, five daily test sessions of unisensory and multisensory performance were conducted using the same methods as in preexposure testing (48–50 trials/location/condition/animal). Visual stimuli in experiment 2 were now tested with low intensity. All testing was performed on both sides of space concomitantly with all trial types pseudo-randomly interleaved. However, the results are described separately for the two hemifields for the two experiments, as different effects were predicted and identified.

#### Data analysis

Responses on stimulus-containing trials were scored as “On-stimulus” if the animal approached the stimulus location, “Off-stimulus” if it approached a different location, and “No-Go” if it remained at the start position (On- and Off-stimulus are both “Go” responses). When trials contained a target stimulus, On-stimulus responses were scored as correct, and all other responses were incorrect. On catch trials, No-Go responses were scored as correct and other responses were scored as incorrect.

To evaluate error patterns, the probability of making a correct response to a target stimulus P[Correct|Stimulus] (aka “performance”) was separated into the product of two conditional probabilities: the probability of making a “Go” response when the stimulus was present (“detection,” P[Go|Stimulus]) and the probability that a “Go” response was to the correct location (“localization,” P[Correct|Go]). A more in-depth description of how these metrics are calculated is available in previous publications (Smyre et al. 2021; Bean et al. 2022). Each metric (performance, detection, and localization) is reported in the text as mean ± SEM averaged over animals within a group and, where convenient, also over test locations.

Multisensory enhancement (ME_SF_) was calculated by comparing the proportionate difference between the response metrics (performance, detection, and localization) in multisensory conditions to those calculated from a “statistical facilitation” (SF) model. This model (consistent with probability summation) represents an assumption that the more accurate of the two modalities (visual or auditory) determined the response on a trial-by-trial basis (Wang et al. 2020; Smyre et al. 2021; Bean et al. 2022). Its predictions were generated using a bootstrapping procedure and tested for each animal and location within an experimental block. An SF prediction for a single trial was generated by randomly selecting a visual-alone and an auditory-alone trial from the dataset and selecting the “better” of the two: the response closest to the target location or, for trials containing only nontarget stimuli, the response closest to the animal’s preferred response location for that stimulus. Any “Go” response was “better” than a “No-Go” response. For each location and visual–auditory combination, this procedure was repeated a number of times equal to the actual number of experimental visual–auditory test trials to create one set of SF predictions for that location/combination over the entire experiment. SF predictions for each summary response metric (e.g. performance) were calculated from this set. Due to the random selection aspect, each run of the procedure can generate different predicted values. To generate distributions for each response metric predicted by SF (i.e. SF-predicted sampling distributions), the procedure was repeated 10,000 times, generating 10,000 sets of SF-predicted experimental results and 10,000 SF predictions for each response metric. Empirical values for each metric calculated over the collected data were directly compared to these SF-predicted sampling distributions. Significance (*p*-values) for multisensory enhancement were estimated from these comparisons for each location and reported in summary by group as mean ± SEM.

A linear mixed effects model (fixed effects of experimental block; random effects of animal and location) was used to compare results between experiments. Maximum likelihood estimation (MLE) was used to quantify regression coefficients for the fixed effect (Δ between conditions). Significance was determined with likelihood ratio tests (adjusted DF method: Kenward-Roger). Linear regressions (F-tests) were used to evaluate significant changes over time for stimulus conditions for each experimental block. A z-test was used to compare ME_SF_ between target locations. Alpha for each analysis was 0.05.

## Results

**Fig. 2:**
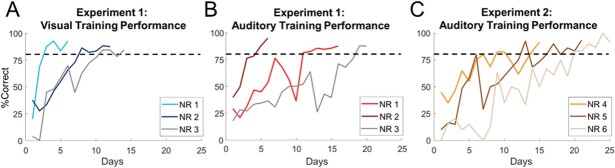
**Learning the task.** Shown is the performance of each animal (NR1–3) across days while learning the visual (**A**) and auditory (**B**) tasks in experiment 1 and the auditory (**C**) task in experiment 2. Data points are averaged across all tested locations and the dashed line represents the performance criterion (80% correct).

Animals in experiment 1 (*n* = 3) were initially trained to approach the location of brief visual and auditory stimuli (“redundant targets” paradigm), while animals in experiment 2 (*n* = 3) were trained to only approach auditory stimulus locations (“auditory targets” paradigm, see Methods). Animals in both cohorts reached the training criterion (>80% correct response rate) rapidly within similar time frames (2–3 weeks), despite their lack of early life experience with patterned auditory stimuli (Fig. 2). It was therefore necessary to reduce the intensity of target stimuli to ensure that unisensory performance was not at ceiling when evaluating the multisensory computation.

### The default multisensory computation was competitive in both experiments:

**Fig. 3:**
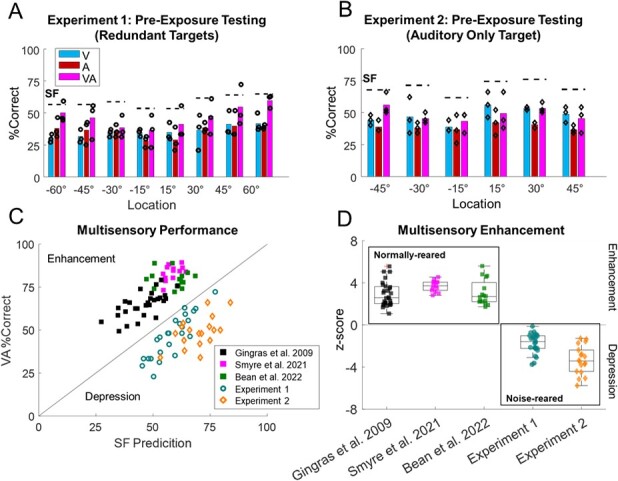
**No multisensory enhancement was exhibited in the task before cross-modal exposure. A/B)** Histograms show group preexposure performance (circles = individual data) in response to visual (V), auditory (A), and cross-modal (VA) stimuli in experiment 1 (A) and 2 (B). Note that VA performance never exceeded and was often significantly lower than SF (dashed horizontal lines). **C)** Scatter plot shows the overall VA performance vs SF predictions for normally-reared animals (filled squares) from Gingras et al. 2009; Smyre et al. 2021; and Bean et al. 2022, as well as for noise-reared animals in the present experiments trained on the redundant targets task in experiment 1 (open circles), and the auditory only target in experiment 2 (open diamond). **D)** Boxplots show the z-score of each animal’s response by location to the cross-modal stimulus vs the predictions of SF. The central line of the box indicates the mean, and the bottom and top edges the 25th and 75th percentiles. The whiskers extend to the extreme data points. Note the contrasting performance of the groups: Enhancement in normally-reared, and depression in noise-reared, animals.

Spatiotemporally congruent visual–auditory stimulus pairs have previously been shown to elicit enhanced detection and localization performance in normally-reared animals (Stein et al. 1989; Stein and Meredith 1993; Burnett et al. 2004, 2007; Rowland et al. 2007b; Gingras et al. 2009;Smyre et al. 2021 ; Bean et al. 2022). But in the animals reared here without normal visual–auditory experience, such stimuli failed to enhance detection, localization, or overall performance (Fig. 3 see Supplementary Tables 1 and 2). The low level of performance (% of correct approaches to the target, see Methods) compared to the predictions of SF indicated that a mutually inhibitory, or “competitive” computation was engaged (Yu et al. 2019). This multisensory “defect” remained unchanged during the initial testing blocks despite the necessary exposure to the congruent visual–auditory test stimuli (experiment 1: R^2^ = 0.2580, *P* = 0.1627; experiment 2: R^2^ = 0.2173, *P* = 0.1745). Similar impairments were observed across the two cohorts (Supplementary Tables 1, 2, 4, and 5), indicating that this phenomenon is robust regardless of the specific training paradigm, stimulus duration, or apparatus used to test it.

Animals trained with auditory targets (experiment 2) were also tested with spatially incongruent visual–auditory pairs in which the visual stimulus was 30° eccentric to the auditory target. These disparate cross-modal stimuli generally depress performance in normally reared animals (Stein et al. 1989; Jiang et al. 2002), and also did so in the animals studied here, lowering overall performance (VA: 39 ± 4.6% vs. SF: 44 ± 2.1%, ME_SF_ = −10 ± 9.8%, *P* = 0.0002), lowering detection probabilities (*P* < 0.001), and increasing localization errors (*P* < 0.001; see Supplementary Tables 6 and 7). As expected (Stein et al. 1989; Jiang et al. 2002; Wallace et al. 2004b; Rowland et al. 2007b), localization errors were significantly (*P* = 0.0044) biased [19 ± 2.8°] towards the 30° eccentric visual stimulus (Supplementary Table 9).

Thus, in the absence of visual–auditory experience, animals processed spatially congruent and spatially disparate visual–auditory stimulus pairs in similar ways. Both configurations induced what appeared to be a competition that led to impairments in detection, localization, and overall performance. These behavioral observations are consistent with those of other studies (Smyre et al. 2021; Bean et al. 2022) and with prior evidence that competitive interactions are a default multisensory computation in SC neurons (Yu et al. 2019). The changes induced by specific experience described below are best understood relative to this initial “default” competitive state that is operational in the absence of normal visual–auditory experience.

## Experiment 1

### Effects of spatially congruent cross-modal exposure

Animals in experiment 1 received 14 sessions involving exposure to spatially congruent visual–auditory stimuli at a single location in one hemifield (left) as described in Methods (A-45°/V-45°) and exposure to the individual modality-specific components (A45°, V45°) at the homotopic location in the opposite hemifield. They were tested intermittently between exposure days with visual and auditory stimuli presented individually and together at multiple spatial positions. At the exposure location on the left, there were no significant unisensory changes in visual (R^2^ = 0.4409, Slope = 0.5871, *P* = 0.1504) or auditory (R^2^ = 0.1005, Slope = −0.197, *P* = 0.5403) performance measures across testing days. However, spatially congruent visual–auditory pairs quickly (within 360 trials, ~6 sessions) began to elicit enhanced performance at the exposure location (VA: 83 ± 4.8% vs. SF: 61 ± 8.1%, ME_SF_ = 39 ± 19%, *P* = 0.0008).

**Fig. 4:**
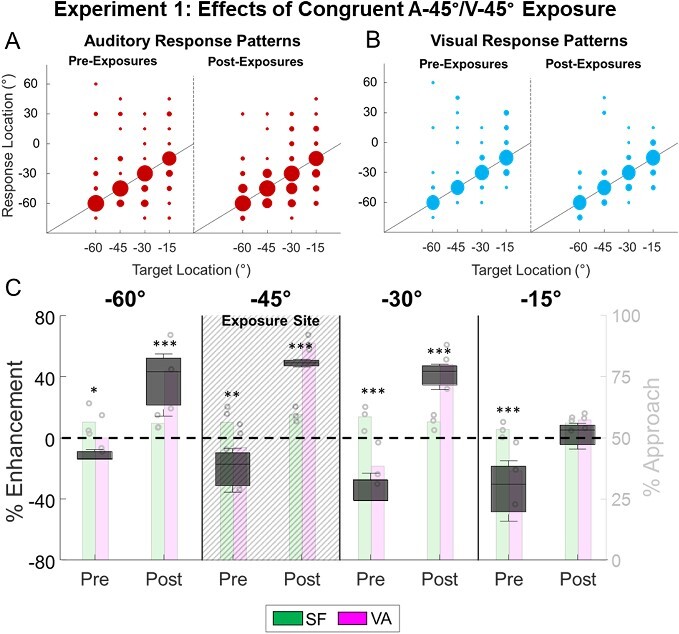
**Exp. 1: Multisensory enhancement after cross-modal exposure. A/B)** Scatterplot shows the response patterns for animals on auditory **(A)** and visual **(B)** trials during preexposure (left) and postexposure (right) testing. For convenience data are collapsed across animals and shown in the left hemifield, and the area of each filled circle is proportional to the number of responses made to that location. Note the similar performance before and after cross-modal exposure. **C)** Black boxplots show the magnitude of the multisensory enhancement (ME_SF_) relative to SF for each location after cross-modal exposure. Background histograms show group multisensory performance (circles show individual performance) and SF predictions (horizontal lines). Note that prior to exposures (pre) VA performance never exceeded, and was often significantly lower, than SF. However, after exposure multisensory performance at or adjacent to the site of exposure (dashed shaded area) significantly exceeded SF. Asterisks indicate significance at different levels (^*^ = *P* < 0.05, ^*^^*^ = *P* < 0.01, ^*^^*^^*^ = *P* < 0.001).

After 14 exposure sessions, unisensory and multisensory responses were evaluated across the visual field. Visual and auditory Off-target localization errors were centered around actual target locations (i.e. they were unbiased), which is typical in this preparation (Fig. 4, see (Stein et al. 1989; Jiang et al. 2002; Rowland, Stanford, et al. 2007; Bean et al. 2021; Smyre et al. 2021)). At the exposure location, multisensory enhancement was now evident in detection (*P* < 0.001), localization (*P* < 0.001), and total performance (ME_SF_ (A-45°/V-45°) = 49 ± 0.9%, *P* < 0.001, Fig. 4C). Similar, but smaller magnitudes of enhancement were observed at nearby locations (ME_SF_ (A-60°/V-60°) =38 ± 7.4%, *P* < 0.001; ME_SF_ (A-30°/V-30°) = 41 ± 3.0%, *P* < 0.001). However, no significant multisensory enhancement in detection (*P* = 0.9259), localization (*P* = 0.2265), or performance (*P* = 0.6064) was evident at the more distant location (−15°) in this hemifield (Supplementary Table 1).

### Effects of modality-specific exposure

**Fig. 5:**
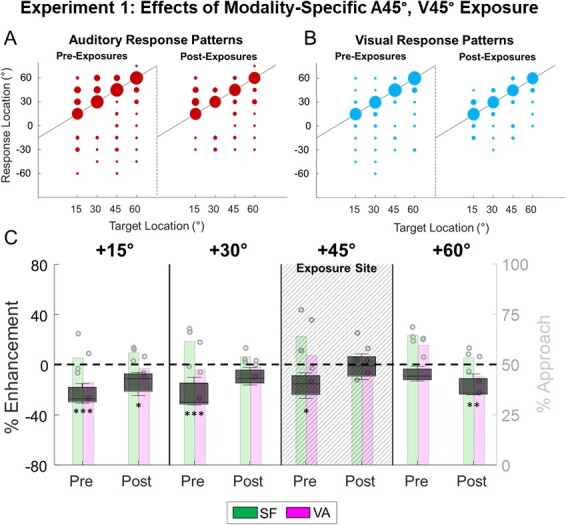
**Exp. 1: No multisensory enhancement after modality-specific exposure. A/B)** Conventions are the same as Fig. 4. Note the similar pre and post exposure unisensory response patterns. **C)** Multisensory performance never exceeded and was often significantly lower than SF both before and after modality-specific exposure. This reveals that the competitive computation was retained at nonexposure sites. ^*^ = *P* < 0.05, ^*^^*^ = *P* < 0.01, ^*^^*^^*^ = *P* < 0.001.

Interleaved with the congruent visual–auditory exposure in the left hemifield, these animals received modality-specific exposure to visual and auditory stimuli at the homotopic locus (A45°, V45°) in the right hemifield. Off-target responses to these modality-specific stimuli were, as to stimuli in the left hemifield, unbiased (Fig. 5A/B). No multisensory enhancement was evident after modality-specific exposure at any tested location (see Fig. 5C, Supplementary Table 2), but performance at the exposure location was consistent with SF (ME_SF_ (+45°) = −1.3 ± 3.6%, *P* = 0.1954).

The results of this experiment reinforce the notion that repeated exposure to congruent visual–auditory exposure leads to the development of visual–auditory enhancement capabilities at and near the exposure location, thereby transforming the multisensory computation from an initial mode of competition to one of cooperation. Modality-specific exposure (in which stimuli are presented independently) also removes the competitive computation but does not confer enhancement capabilities. The visual and auditory signals are now treated as independent and noncompetitive so that ME_SF_ values at their exposure location are not significantly different from zero (*P* = 0.1954).

## Experiment 2

### Effects of spatially congruent cross-modal exposure

Animals in experiment 2 received the same exposure to a spatially congruent combination of highly effective visual–auditory stimuli at a single location in one hemifield (left) as in Exp 1 (A-45°/V-45°). Because these animals were trained in the “auditory targets” paradigm, the correct response during testing was to approach the auditory stimulus even in the presence of a spatially disparate visual stimulus.

**Fig. 6:**
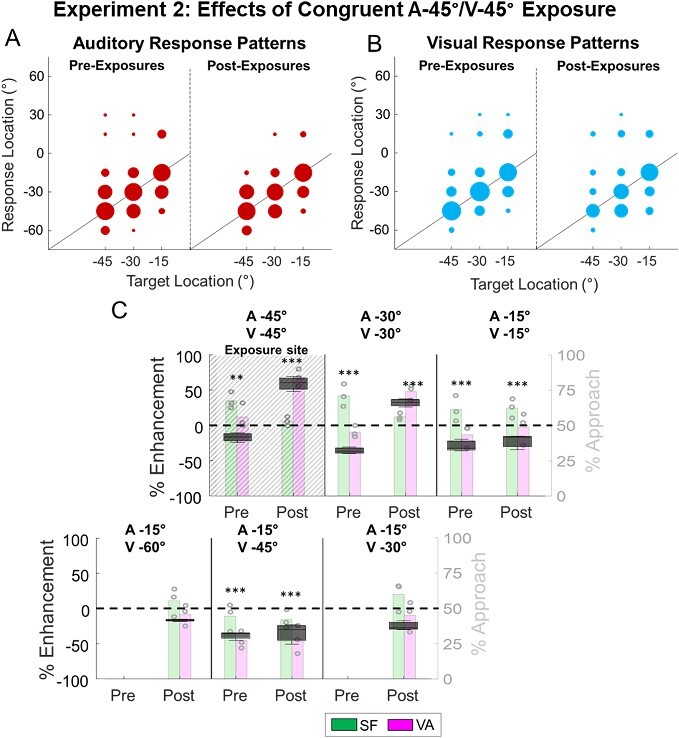
**Multisensory enhancement at the congruent cross-modal exposure site, and competition at more distant sites. A/B)** Conventions are the same as in Fig. 4. Note that as in Exp 1 the auditory and visual response patterns were largely unchanged by the exposure trials. **C)** After exposure to spatially congruent cross-modal cues, multisensory (VA) performance to this configuration was enhanced at the exposure site and at the adjacent site. At more distant sites, however, multisensory performance was below SF revealing the retention of the competitive multisensory computation there. ^*^ = *P* < 0.05, ^*^^*^ = *P* < 0.01, ^*^^*^^*^ = *P* < 0.001.

The results in the left hemifield were consistent with those in Exp. 1, despite differences in the training paradigm, stimulus duration, and apparatus (Fig. 6). Off-target responses to individual stimuli were again unbiased (Fig. 6A, B). Post-exposure testing revealed that multisensory enhancement in detection (*P* < 0.0001), localization (*P* < 0.001), and total performance (ME_SF_(A-45°/V-45°) = 59 ± 6.1%, *P* < 0.001, see Fig. 6C, Supplementary Table 4) had developed at the exposure location. Somewhat lower enhancement was also present at a nearby site (ME_SF_(A-30°/V-30°) =32 ± 3.3%, *P* < 0.001), but not at more distant locations (ME_SF_ (A-15°/V-15°) = −23 ± 4.3%, *P* = 0.0005). The multisensory enhancement magnitude at the exposure location was comparable to that observed at the exposure location in Exp. 1 (*P* = 0.2376). The competitive interactions elicited by spatially disparate stimuli did not appear to be affected by any of the visual–auditory exposure configurations (congruent within the hemifield, disparate exposure in the opposite hemifield) (Fig. 6C, Supplementary Tables 4 and 6, see below).

### Experiment 2: effects of spatially disparate cross-modal exposure:

**Fig. 7:**
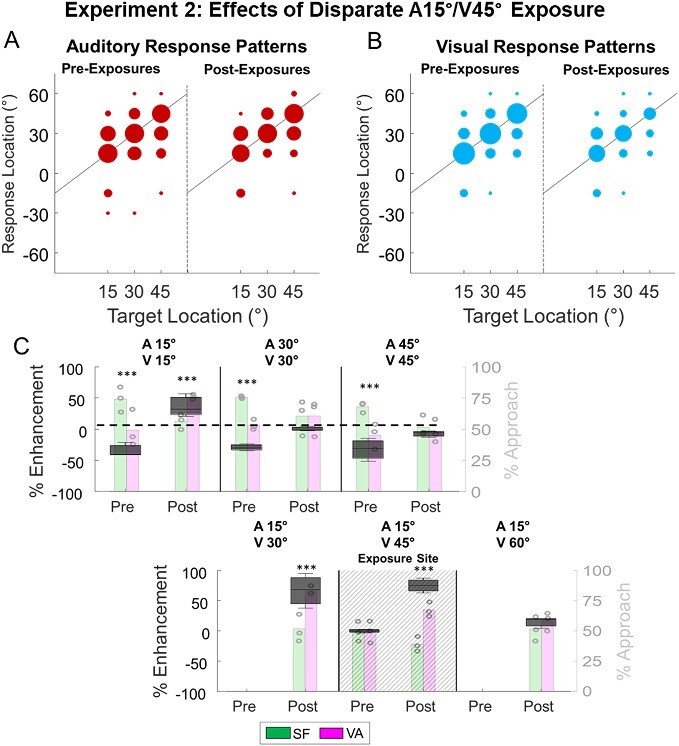
**After exposure to disparate cross-modal cues, multisensory processing principles generalized to disparity combinations contained within that region. A/B)** Conventions here are the same as in previous figures. Note that repeated exposure to the spatially disparate configuration did not alter unisensory response biases. **C)** After disparate cross-modal exposure, multisensory performance exceeded SF predictions to the exposure configuration (A15°/V45°) and to configurations within the exposure region (A15°/V30°, A15°/V15°). Note that multisensory enhancement was not elicited by reversing the locations of a and V (i.e. A45°/V15°) indicating that the principle developed was linked to the configuration of a & V and not to their physical disparity per se. Similarly, their coincidence outside the exposure area failed to elicit enhancement. ^*^ = *P* < 0.05, ^*^^*^ = *P* < 0.01, ^*^^*^^*^ = *P* < 0.001.

In the right hemifield animals were exposed to a visual–auditory configuration with 30° of spatial disparity (A15°/V45°). As in Exp. 1, they were tested with trials interleaved with all stimulus configurations. Notably, the disparate exposure paradigm did not change the pattern of Off-target responses to modality-specific stimuli, which remained unbiased (*P* = 0.1117, Fig. 7A). Thus, exposure to this disparate configuration did not shift auditory localization. However, the effects on multisensory responses were complex and require detailed explanation.

The exposure configuration elicited enhanced detection (*P* < 0.001), localization (*P* < 0.001), and overall performance (*P* < 0.001) to the disparate stimulus configuration. Thus, the simultaneous but spatially disparate visual stimulus increased the rate of correct approaches to the auditory target by ~ 75% (ME_SF_ = 75 ± 4.9%, *P* < 0.001, Fig. 7C, Supplementary Table 7). The magnitude of the enhancement to the disparate configuration at its exposure location on the right approximated that elicited by spatiotemporally congruent stimuli at its exposure location on the left (*P* = 0.7042) described above. This required a substantial change from the depressed multisensory responses elicited by this configuration that was elicited prior to exposure (Pre vs. Post ΔME_SF_: 67 ± 18.9%; *P* = 0.0002).

This response enhancement extended to spatial disparities that were within (and thus smaller than) the exposure configuration. Thus, A15°/V30° showed enhancement (albeit, marginal) in detection (*P* = 0.0657), and significant enhancement in localization (*P* = 0.0039) and overall performance (*P* < 0.001). Its enhancement was similar (*P* = 0.6205) to that elicited by the exposure configuration (ME_SF_ (A15°/V30°) = 67 ± 10%) and also to the enhancement observed for the congruent configuration after congruent exposure on the opposite side of space (suggesting it was at ceiling). When the visual–auditory stimulus disparity was reduced to zero (A15°/V15°), it also elicited enhanced detection (*P* = 0.0073), localization (*P* < 0.001), and overall performance (*P* < 0.001). However, the level of enhancement was significantly lower (*P* < 0.001) than that elicited by the exposure configuration (ME_SF_(A15°/V15°) = 36 ± 7.6). In each of these configurations, the location of the auditory stimulus matched that of the exposure condition (i.e. 15°). No other congruent visual–auditory stimuli in the right hemisphere elicited enhanced responses (Fig. 7, Supplementary Tables 5 and 7).

It is important to note that all other spatially disparate cues tested produced responses consistent with or below SF (Supplementary Table 7). This included a configuration with the same auditory location as the exposure stimulus but a greater spatial disparity (ME_SF_(A15°/V60°) = 14 ± 3, *P* = 0.1998) and a configuration that flipped the auditory and visual locations from the exposure stimulus (ME_SF_(A45°/V15°) = −26 ± 8.1, *P* = 0.0039).

## Discussion

*Competition, Statistical Facilitation, and Cooperation*. The present study examined how adult noise-reared animals develop the ability to use information from different senses (i.e. visual, and auditory) synergistically. Noise-rearing restricts animals’ exposure to patterned auditory and (crucially, here) their spatiotemporal pairing with visual stimuli. The effects of sensory restriction on subsequent sensory processing in the adult have been previously evaluated in both audition (Tees 1967; Chang and Merzenich 2003; Amaral et al. 2024) and vision (Chalupa et al. 1978; Buisseret and Gary-Bobo 1979; Lund and Mitchell 1979; Benevento et al. 1992; Morales et al. 2002; Wallace et al. 2004a; Carrasco et al. 2011; Yu et al. 2013). These experimental manipulations resemble naturally-occurring developmental abnormalities such as congenital deafness (Kral et al. 2002; Butler and Lomber 2013) and congenital cataracts (Putzar et al. 2007, 2010; Senna et al. 2022; Singh et al. 2022), both of which can often be ameliorated later in life through surgical intervention. Here, any rearing-induced effects on visual and/or auditory sensitivity were offset by comparable adjustments of stimulus intensity in order to directly assess the effects on multisensory integration.

Multisensory integration is a critical feature of normal perception and a common biological invention for enhancing the salience of informative sensory signals. In many circumstances the coincident activation of multiple senses mitigates ambiguity, producing more rapid, reliable, and robust perception and behavior. But to create this “normal” cooperative multisensory platform, the brain must override its default computation of multisensory competition which is operational in the current animal model before visual–auditory experience is acquired (Cuppini et al. 2011, 2012; Bottari et al. 2016; Yu et al. 2019; Smyre et al. 2021; Bean et al. 2022). We hypothesize that repeated congruent exposures promotes the emergence of a cooperative interaction that overrides the expression of the “default” competition (Yu et al. 2019).

The default competitive computation is suboptimal when applied to spatiotemporally congruent cross-modal stimuli in all contexts in which they are mutually informative. This includes the cross-modal events common in “normal” environments and in the specific task engaged here. Prior to the cross-modal exposure blocks in these experiments, animals’ performance was poorer than if they simply used the most reliable unisensory signal on each trial (i.e. SF or probability summation). However, this apparent competition induced by multiple stimuli (cross-modal in this context) is not an uncommon or unlikely neural computation: it is inherent to decision-making. In the present tasks animals must select a single location for each response and, in the absence of prior information that the visual and auditory signals derive from a common target (even when they are at the same location), the signals are competitors for control of the underlying sensorimotor architecture.

The present study illustrates that the competitive computation of the naïve multisensory brain can be overridden by sensory exposure that communicates reliable relationships between cross-modal sensory signals. For example, exposure trials in which visual and auditory stimuli were repeatedly presented individually and not together communicated that the signals were independent of one another, and the animals adapted their multisensory behavior accordingly by adopting a computation consistent with independent processing, specifically, SF. In contrast, exposure trials in which visual–auditory stimuli were repeatedly presented together in fixed relationships communicated that signals in that relationship were mutually informative. Consequently, the animals adapted to process them synergistically reaping performance benefits in these tasks far exceeding predictions of SF. The present data suggest that this process of adaptation is rapid and that thus the trajectories to these endpoints are distinct. However, it is possible that developing the cooperative multisensory computation from the competitive default computation covertly involves (or could be facilitated by) the explicit inclusion of a transitory stage in which the signals are processed independently.

### Physiological-behavioral parallels

Although the specific microcircuit changes induced by visual–auditory experience that alter the initial competitive design are not known, their physiological impact has been well-documented at the level of the single SC neuron. There are striking parallels in the developmental trajectories, constraints, and contingencies observed here in multisensory behavior and changes observed in these individual multisensory neurons (Wallace and Stein 2007; Yu et al. 2010; Stein et al. 2014; Xu et al. 2014, 2015, 2017; Wang et al. 2020). As seen in the behavior examined here, multisensory SC neurons also engage in competitive multisensory computations by default (Yu et al. 2019). When “naïve” adult SC neurons are exposed to invariant spatiotemporally congruent visual–auditory stimuli, they soon develop the capability to enhance responses to these and other congruent visual–auditory stimuli (Yu et al. 2010; Xu et al. 2017), an effect observed here in behavioral performance. Similarly, this synergistic computation is not induced by exposure to the component stimuli individually and is not generalized across space. Only neurons with receptive fields encompassing the exposure location are affected, others retain their competitive computation (Yu et al. 2010). This physiological effect is constrained to the region of space in which cross-modal experience was gathered just as was the behavioral effects observed here. In addition, repeated exposure to spatially disparate visual–auditory stimuli causes individual SC neurons to integrate disparate stimuli to produce enhanced responses—the neural correlate of the behavioral reaction to such cues that was observed here (Wallace and Stein 2007).

These neuronal–behavioral parallels are unlikely to be coincidental, as the multisensory aspects of the behaviors examined here are known to involve SC neurons (Burnett et al. 2004, 2007). However, they are nonetheless striking because these overt, coordinated whole-body orientation behaviors engage a host of unisensory and multisensory circuits (Grillner et al. 2008; Pernía et al. 2021), each of which likely follows its own developmental trajectory and constraints. The present results suggest that either the circuitry of the SC is dominant in controlling the multisensory computation evident in these behaviors, or that other participating regions respond to these cross-modal experiences in similar ways.

The physiology of multisensory SC development may also provide some insight into the behavioral adaptability noted in the present study. There exists a rough topographic alignment of the different sensory representation in the SC long before multisensory integration is possible (Stein et al. 1973, 2014; Norton 1974; Meredith and Stein 1990; Withington-Wray et al. 1990; Wallace et al. 1997; Wallace and Stein 1997; Royal et al. 2010). Although the constituent receptive fields of these neurons are initially large, they are in rough alignment with one another and with the underlying motor map. These circuit features favor adaptation to cross-modal stimuli within a limited region of spatial correspondence and help explain the effect of cross-modal exposure to locations at and near the exposure site.

The architecture of the underlying circuit can also be used to predict relationships not yet studied. For example, it is likely that parallels will be found in the development of behaviors involving other sensory cue combinations, specifically those involving tactile cues. The SC somatosensory representation has a topography that is in register with its visual and auditory counterparts, each of the possible multisensory convergence patterns is present in its population, and all multisensory interactions among these modalities normally follow similar integrative principles (Dräger and Hubel 1975, 1976; Stein et al. 1975, 1983; Chalupa and Rhoades 1977; Stein 1978; Clemo and Stein 1986; Meredith and Stein 1986b; Meredith et al. 1992; Stein and Meredith 1993; Wallace et al. 1993; Benedetti 1995; Groh and Sparks 1996; Benedek et al. 1997; Triplett et al. 2012; Xu et al. 2015).

### Developing multiple principles for integrating the same cross-modal cues

**Fig. 8:**
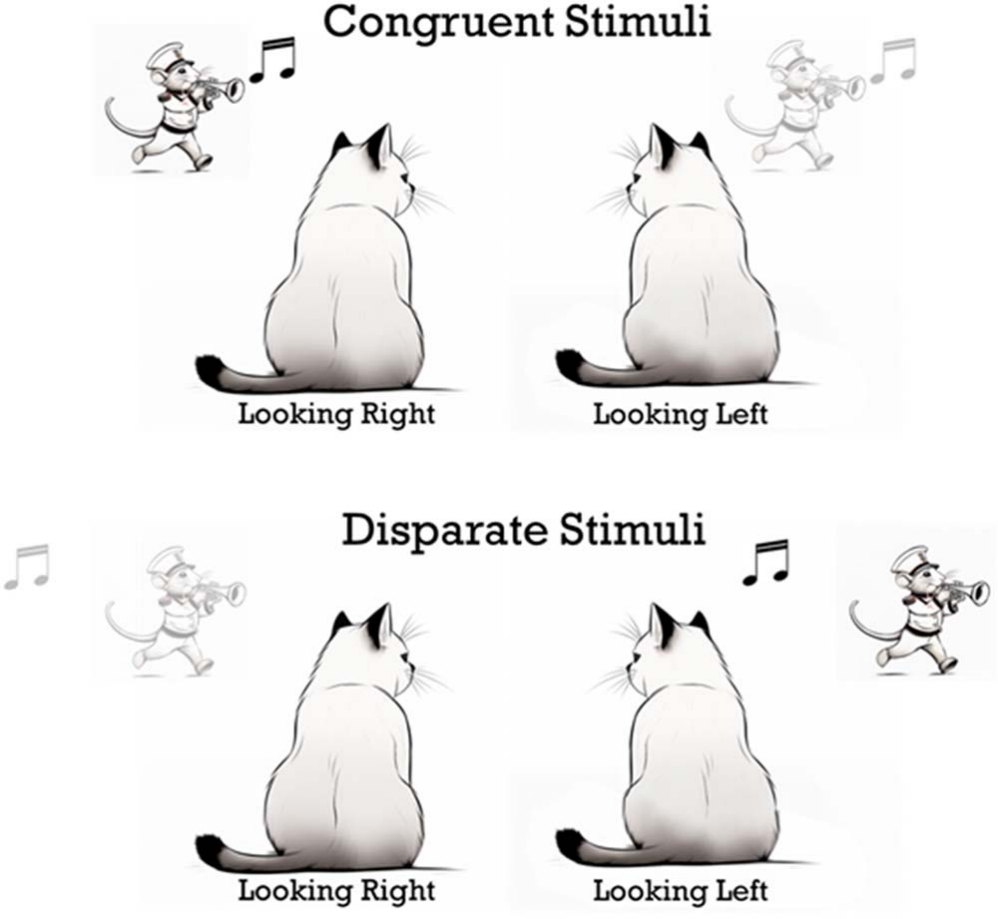
**Conflicting principles of multisensory integration can coexist:** Shown are likely consequences of the rearing conditions in Exp. 2. Naïve animals were exposed to a visual–auditory stimulus that was spatially congruent on the left and spatially disparate on the right. **top:** The same event, illustrated here as the sight and sound of a mouse playing a trumpet, is enhanced when the cat looks to the right, but depressed when it looks to the left. **bottom:** Rendering those component stimuli disparate leads to the event being depressed when the animal looks right, but enhanced when it looks left, see text for a more detailed discussion.

Another straightforward prediction from physiological studies was that, given the spatial limits of SC receptive fields and the specificity of their experience-based changes, the system should have the ability to encode multiple and even conflicting processing rules. It does, and this was what the results of the current behavioral studies demonstrated in animals receiving different cross-modal experiences in the two hemispheres. There is little doubt that the same effect would be induced in the same hemisphere if those configurations were sufficiently separated from one another. Nevertheless, it seems counterintuitive that the totality of experience would not be used to create a single generic template that could be used to enhance responses to spatiotemporally concordant cross-modal cues regardless of their location. Indeed, these results seem anomalous from the perspective of normal development, suggesting that for these animals visual–auditory events would be processed in qualitatively different ways when it was to the right or left of the observer as shown in Fig. 8.

The stages of multisensory development noted here may also help understand recent observations in studies of human patients whose visual or auditory processing was compromised early in life (e.g. congenital cataracts, or congenital deafness). They too may retain an initial default competitive multisensory computation, which may explain why their processing of conflicting cross-modal stimuli can appear intact (Bruns and Röder 2023). Correcting these deficits (e.g. by replacement of the lens or a cochlear implant) coupled with sufficient experience with congruent cross-modal events eventually leads to the development of more typical multisensory integration capabilities, although residual defects may persist (Nava et al. 2014; Stevenson et al. 2017; Senna et al. 2021; Bruns and Röder 2023). Variation in the incidence and rate of “spontaneous” recovery in such individuals may be attributable to variations in the quality and density of those experiences. The results here suggest the possibility of enhancing the speed of that later developmental process, for greater therapeutic effectiveness, by directly manipulating experience (Cappagli et al. 2017; Senna et al. 2021, 2022). Thus, they provide another context in which cross-modal exposure paradigms can be applied to benefit perceptual function (Finocchietti et al. 2015; Jiang et al. 2015, 2020, 2021; Dakos et al. 2019, 2020; Bean et al. 2023; Rowland et al. 2023).

## Supplementary Material

Supplementary_Tables_bhae247

## References

[ref1] Amaral L , Wang X, Bi Y, Striem-Amit E. Unraveling the impact of congenital deafness on individual brain organization. elife. 2024:13. 10.7554/eLife.96944 PMC11903032 40072311

[ref2] Bean NL , Stein BE, Rowland BA. Stimulus value gates multisensory integration. Eur J Neurosci. 2021:53(9):3142–3159. 10.1111/ejn.15167. 33667027 PMC8177070

[ref3] Bean NL , Smyre SA, Stein BE, Rowland BA. Noise-rearing precludes the behavioral benefits of multisensory integration. Cereb Cortex. 2022:33(4):948–958. 10.1093/cercor/bhac113. PMC9930622 35332919

[ref4] Bean NL , Stein BE, Rowland BA. Cross-modal exposure restores multisensory enhancement after hemianopia. Cereb Cortex. 2023:33(22):11036–11046. 10.1093/cercor/bhad343. 37724427 PMC10646694

[ref5] Benedek G , Perény J, Kovács G, Fischer-Szátmári L, Katoh YY. Visual, somatosensory, auditory and nociceptive modality properties in the feline suprageniculate nucleus. Neuroscience. 1997:78(1):179–189. 10.1016/S0306-4522(96)00562-3. 9135099

[ref6] Benedetti F . Differential formation of topographic maps on the cerebral cortex and superior colliculus of the mouse by temporally correlated tactile- tactile and tactile-visual inputs. Eur J Neurosci. 1995:7(9):1942–1951. 10.1111/j.1460-9568.1995.tb00717.x. 8528470

[ref7] Benevento LA , Bakkum BW, Port JD, Cohen RS. The effects of dark-rearing on the electrophysiology of the rat visual cortex. Brain Res. 1992:572(1–2):198–207. 10.1016/0006-8993(92)90470-T. 1611513

[ref8] Bottari D , Troje NF, Ley P, Hense M, Kekunnaya R, Röder B. Sight restoration after congenital blindness does not reinstate alpha oscillatory activity in humans. Sci Rep. 2016:6(1):24683. 10.1038/srep24683. 27080158 PMC4832338

[ref9] Bruns P , Röder B. Development and experience-dependence of multisensory spatial processing. Trends Cogn Sci S1364-6613(23)00102-X. 2023:27(10):961–973. 10.1016/j.tics.2023.04.012. 37208286

[ref10] Buisseret P , Gary-Bobo E. Development of visual cortical orientation specificity after dark-rearing: role of extraocular proprioception. Neurosci Lett. 1979:13(3):259–263. 10.1016/0304-3940(79)91504-0. 530478

[ref11] Burnett LR , Stein BE, Chaponis D, Wallace MT. Superior colliculus lesions preferentially disrupt multisensory orientation. Neuroscience. 2004:124(3):535–547. 10.1016/j.neuroscience.2003.12.026. 14980725

[ref12] Burnett LR , Stein BE, Perrault TJ Jr, Wallace MT. Excitotoxic lesions of the superior colliculus preferentially impact multisensory neurons and multisensory integration. Exp Brain Res. 2007:179(2):325–338. 10.1007/s00221-006-0789-8. 17146648

[ref13] Butler BE , Lomber SG. Functional and structural changes throughout the auditory system following congenital and early-onset deafness: implications for hearing restoration. Front Syst Neurosci. 2013:7:92. 10.3389/fnsys.2013.00092. 24324409 PMC3840613

[ref14] Cao Y , Summerfield C, Park H, Giordano BL, Kayser C. Causal inference in the multisensory brain. Neuron. 2019:102(5):1076–1087.e8. 10.1016/j.neuron.2019.03.043. 31047778

[ref15] Cappagli G , Finocchietti S, Baud-Bovy G, Cocchi E, Gori M. Multisensory rehabilitation training improves spatial perception in totally but not partially visually deprived children. Front Integr Neurosci. 2017:11:29. 10.3389/fnint.2017.00029. 29097987 PMC5654347

[ref16] Carrasco MM , Mao Y-T, Balmer TS, Pallas SL. Inhibitory plasticity underlies visual deprivation-induced loss of receptive field refinement in the adult superior colliculus. Eur J Neurosci. 2011:33(1):58–68. 10.1111/j.1460-9568.2010.07478.x. 21050281 PMC4940179

[ref17] Chalupa LM , Rhoades RW. Responses of visual, somatosensory, and auditory neurones in the golden hamster’s superior colliculus. J Physiol Lond. 1977:270(3):595–626. 10.1113/jphysiol.1977.sp011971. 903907 PMC1353534

[ref18] Chalupa LM , Morrow AL, Rhoades RW. Behavioral consequences of visual deprivation and restriction in the golden hamster. Exp Neurol. 1978:61(2):442–454. 10.1016/0014-4886(78)90259-5. 710559

[ref19] Chang EF , Merzenich MM. Environmental noise retards auditory cortical development. Science. 2003:300(5618):498–502. 10.1126/science.1082163. 12702879

[ref20] Clemo HR , Stein BE. Effects of cooling somatosensory cortex on response properties of tactile cells in the superior colliculus. J Neurophysiol. 1986:55(6):1352–1368. 10.1152/jn.1986.55.6.1352. 3734860

[ref21] Collignon O , Dormal G, de Heering A, Lepore F, Lewis TL, Maurer D. Long-lasting Crossmodal cortical reorganization triggered by brief postnatal visual deprivation. Curr Biol. 2015:25(18):2379–2383. 10.1016/j.cub.2015.07.036. 26299512

[ref22] Cuppini C , Stein BE, Rowland BA, Magosso E, Ursino M. A computational study of multisensory maturation in the superior colliculus (SC). Exp Brain Res. 2011:213(2–3):341–349. 10.1007/s00221-011-2714-z. 21556818 PMC3235682

[ref23] Cuppini C , Magosso E, Rowland B, Stein B, Ursino M. Hebbian mechanisms help explain development of multisensory integration in the superior colliculus: a neural network model. Biol Cybern. 2012:106(11–12):691–713. 10.1007/s00422-012-0511-9. 23011260 PMC3552306

[ref24] Dakos AS , Walker EM, Jiang H, Stein BE, Rowland BA. Interhemispheric visual competition after multisensory reversal of hemianopia. Eur J Neurosci. 2019:50(11):3702–3712. 10.1111/ejn.14554. 31430406 PMC6928431

[ref25] Dakos AS , Jiang H, Stein BE, Rowland BA. Using the principles of multisensory integration to reverse hemianopia. Cereb Cortex. 2020:30(4):2030–2041. 10.1093/cercor/bhz220. 31799618 PMC7175010

[ref26] Dräger UC , Hubel DH. Physiology of visual cells in mouse superior colliculus and correlation with somatosensory and auditory input. Nature. 1975:253(5488):203–204. 10.1038/253203a0. 1110771

[ref27] Dräger UC , Hubel DH. Topography of visual and somatosensory projections to mouse superior colliculus. J Neurophysiol. 1976:39(1):91–101. 10.1152/jn.1976.39.1.91. 1249606

[ref28] Efrati A , Gutfreund Y. Early life exposure to noise alters the representation of auditory localization cues in the auditory space map of the barn owl. J Neurophysiol. 2011:105(5):2522–2535. 10.1152/jn.00078.2011. 21368005

[ref29] Finocchietti S , Cappagli G, Ben Porquis L, Baud-Bovy G, Cocchi E, Gori M. Evaluation of the audio bracelet for blind interaction for improving mobility and spatial cognition in early blind children—a pilot study. Annu Int Conf IEEE Eng Med Biol Soc. 2015:2015:7998–8001. 10.1109/EMBC.2015.7320248. 26738148

[ref30] Gingras G , Rowland BA, Stein BE. The differing impact of multisensory and unisensory integration on behavior. J Neurosci. 2009:29(15):4897–4902. 10.1523/JNEUROSCI.4120-08.2009. 19369558 PMC2678542

[ref31] Grillner S , Wallén P, Saitoh K, Kozlov A, Robertson B. Neural bases of goal-directed locomotion in vertebrates—an overview. Brain Res Rev. 2008:57(1):2–12. 10.1016/j.brainresrev.2007.06.027. 17916382

[ref32] Groh JM , Sparks DL. Saccades to somatosensory targets. III. Eye-position-dependent somatosensory activity in primate superior colliculus. J Neurophysiol. 1996:75(1):439–453. 10.1152/jn.1996.75.1.439. 8822569

[ref33] Jiang W , Wallace MT, Jiang H, Vaughan JW, Stein BE. Two cortical areas mediate multisensory integration in superior colliculus neurons. J Neurophysiol. 2001:85(2):506–522. 10.1152/jn.2001.85.2.506. 11160489

[ref34] Jiang W , Jiang H, Stein BE. Two corticotectal areas facilitate multisensory orientation behavior. J Cogn Neurosci. 2002:14(8):1240–1255. 10.1162/089892902760807230. 12495529

[ref35] Jiang H , Stein BE, McHaffie JG. Multisensory training reverses midbrain lesion-induced changes and ameliorates haemianopia. Nat Commun. 2015:6(1):7263. 10.1038/ncomms8263. 26021613 PMC6193257

[ref36] Jiang H , Rowland BA, Stein BE. Reversing hemianopia by multisensory training under Anesthesia. Front Syst Neurosci. 2020:14:4. 10.3389/fnsys.2020.00004. 32076401 PMC7006460

[ref37] Jiang H , Stanford TR, Rowland BA, Stein BE. Association cortex is essential to reverse hemianopia by multisensory training. Cereb Cortex. 2021:31(11):5015–5023. 10.1093/cercor/bhab138. 34056645 PMC8491673

[ref38] Kayser C , Shams L. Multisensory causal inference in the brain. PLoS Biol. 2015:13(2):e1002075. 10.1371/journal.pbio.1002075. 25710476 PMC4339834

[ref39] Körding KP , Beierholm U, Ma WJ, Quartz S, Tenenbaum JB, Shams L. Causal inference in multisensory perception. PLoS One. 2007:2(9):e943. 10.1371/journal.pone.0000943. 17895984 PMC1978520

[ref40] Kral A , Hartmann R, Tillein J, Heid S, Klinke R. Hearing after congenital deafness: central auditory plasticity and sensory deprivation. Cereb Cortex. 2002:12(8):797–807. 10.1093/cercor/12.8.797. 12122028

[ref41] Lund RD , Mitchell DE. The effects of dark-rearing on visual callosal connections of cats. Brain Res. 1979:167(1):172–175. 10.1016/0006-8993(79)90273-7. 455063

[ref42] Meredith MA , Stein BE. Interactions among converging sensory inputs in the superior colliculus. Science. 1983:221(4608):389–391. 10.1126/science.6867718. 6867718

[ref43] Meredith MA , Stein BE. Spatial factors determine the activity of multisensory neurons in cat superior colliculus. Brain Res. 1986a:365(2):350–354. 10.1016/0006-8993(86)91648-3. 3947999

[ref44] Meredith MA , Stein BE. Visual, auditory, and somatosensory convergence on cells in superior colliculus results in multisensory integration. J Neurophysiol. 1986b:56(3):640–662. 10.1152/jn.1986.56.3.640. 3537225

[ref45] Meredith MA , Stein BE. The visuotopic component of the multisensory map in the deep laminae of the cat superior colliculus. J Neurosci. 1990:10(11):3727–3742. 10.1523/JNEUROSCI.10-11-03727.1990. 2230957 PMC6570087

[ref46] Meredith MA , Wallace MT, Stein BE. Visual, auditory and somatosensory convergence in output neurons of the cat superior colliculus: multisensory properties of the tecto-reticulo-spinal projection. Exp Brain Res. 1992:88(1):181–186. 10.1007/BF02259139. 1541354

[ref47] Miller RL , Stein BE, Rowland BA. Multisensory integration uses a real-time Unisensory-multisensory transform. J Neurosci. 2017:37(20):5183–5194. 10.1523/JNEUROSCI.2767-16.2017. 28450539 PMC5444199

[ref48] Morales B , Choi S-Y, Kirkwood A. Dark rearing alters the development of GABAergic transmission in visual cortex. J Neurosci. 2002:22(18):8084–8090. 10.1523/JNEUROSCI.22-18-08084.2002. 12223562 PMC6758086

[ref49] Nava E , Bottari D, Villwock A, Fengler I, Büchner A, Lenarz T, Röder B. Audio-tactile integration in congenitally and late deaf cochlear implant users. PLoS One. 2014:9(6):e99606. 10.1371/journal.pone.0099606. 24918766 PMC4053428

[ref50] Norton TT . Receptive-field properties of superior colliculus cells and development of visual behavior in kittens. J Neurophysiol. 1974:37(4):674–690. 10.1152/jn.1974.37.4.674. 4837772

[ref51] Pernía-Andrade AJ , Wenger N, Esposito MS, Tovote P. Circuits for state-dependent modulation of locomotion. Front Hum Neurosci. 2021:15:745689. 10.3389/fnhum.2021.745689. 34858153 PMC8631332

[ref52] Putzar L , Goerendt I, Lange K, Rösler F, Röder B. Early visual deprivation impairs multisensory interactions in humans. Nat Neurosci. 2007:10(10):1243–1245. 10.1038/nn1978. 17873871

[ref53] Putzar L , Hötting K, Röder B. Early visual deprivation affects the development of face recognition and of audio-visual speech perception. Restor Neurol Neurosci. 2010:28(2):251–257. 10.3233/RNN-2010-0526. 20404412

[ref54] Rohe T , Noppeney U. Cortical hierarchies perform Bayesian causal inference in multisensory perception. PLoS Biol. 2015:13(2):e1002073. 10.1371/journal.pbio.1002073. 25710328 PMC4339735

[ref55] Rowland BA , Stein BE. Multisensory integration produces an initial response enhancement. Front Integr Neurosci. 2007:1:4. 10.3389/neuro.07.004.2007. 18958232 PMC2526011

[ref56] Rowland BA , Quessy S, Stanford TR, Stein BE. Multisensory integration shortens physiological response latencies. J Neurosci. 2007a:27(22):5879–5884. 10.1523/JNEUROSCI.4986-06.2007. 17537958 PMC6672269

[ref57] Rowland B , Stanford T, Stein B. A Bayesian model unifies multisensory spatial localization with the physiological properties of the superior colliculus. Exp Brain Res. 2007b:180(1):153–161. 10.1007/s00221-006-0847-2. 17546470

[ref58] Rowland BA , Jiang W, Stein BE. Brief cortical deactivation early in life has long-lasting effects on multisensory behavior. J Neurosci. 2014:34(21):7198–7202. 10.1523/JNEUROSCI.3782-13.2014. 24849354 PMC4028497

[ref59] Rowland BA , Bushnell CD, Duncan PW, Stein BE. Ameliorating hemianopia with multisensory training. J Neurosci JN-RM-0962-22. 2023:43(6):1018–1026. 10.1523/JNEUROSCI.0962-22.2022. 36604169 PMC9908311

[ref60] Royal DW , Krueger J, Fister MC, Wallace MT. Adult plasticity of spatiotemporal receptive fields of multisensory superior colliculus neurons following early visual deprivation. Restor Neurol Neurosci. 2010:28(2):259–270. 10.3233/RNN-2010-0488. 20404413 PMC3652394

[ref61] Senna I , Andres E, McKyton A, Ben-Zion I, Zohary E, Ernst MO. Development of multisensory integration following prolonged early-onset visual deprivation. Curr Biol. 2021:31(21):4879–4885.e6. 10.1016/j.cub.2021.08.060. 34534443

[ref62] Senna I , Piller S, Gori M, Ernst M. The power of vision: calibration of auditory space after sight restoration from congenital cataracts. Proc Biol Sci. 2022:289(1984):20220768. 10.1098/rspb.2022.0768. 36196538 PMC9532985

[ref63] Singh R , Barker L, Chen SI, Shah A, Long V, Dahlmann-Noor A. Surgical interventions for bilateral congenital cataract in children aged two years and under. Cochrane Database Syst Rev. 2022:9:CD003171. 36107778 10.1002/14651858.CD003171.pub3 PMC9477380

[ref64] Smyre SA , Wang Z, Stein BE, Rowland BA. Multisensory enhancement of overt behavior requires multisensory experience. Eur J Neurosci. 2021:54(2):4514–4527. 10.1111/ejn.15315. 34013578 PMC8295226

[ref65] Stein BE . Nonequivalent visual, auditory, and somatic corticotectal influences in cat. J Neurophysiol. 1978:41(1):55–64. 10.1152/jn.1978.41.1.55. 621546

[ref66] Stein BE (Ed.). 2012. The new handbook of multisensory processing. Cambridge, Mass: MIT Press, 10.7551/mitpress/8466.001.0001.

[ref67] Stein BE , Meredith MA. The merging of the senses, cognitive neuroscience series. Cambridge, Mass: MIT Press; 1993.

[ref68] Stein BE , Labos E, Kruger L. Sequence of changes in properties of neurons of superior colliculus of the kitten during maturation. J Neurophysiol. 1973:36(4):667–679. 10.1152/jn.1973.36.4.667. 4713313

[ref69] Stein BE , Magalhaes-Castro B, Kruger L. Superior colliculus: visuotopic-somatotopic overlap. Science. 1975:189(4198):224–226. 10.1126/science.1094540. 1094540

[ref70] Stein BE , Spencer RF, Edwards SB. Corticotectal and corticothalamic efferent projections of SIV somatosensory cortex in cat. J Neurophysiol. 1983:50(4):896–909. 10.1152/jn.1983.50.4.896. 6313872

[ref71] Stein BE , Meredith MA, Huneycutt WS, McDade L. Behavioral indices of multisensory integration: orientation to visual cues is affected by auditory stimuli. J Cogn Neurosci. 1989:1(1):12–24. 10.1162/jocn.1989.1.1.12. 23968407

[ref72] Stein BE , Stanford TR, Rowland BA. Development of multisensory integration from the perspective of the individual neuron. Nat Rev Neurosci. 2014:15(8):520–535. 10.1038/nrn3742. 25158358 PMC4215474

[ref73] Stevenson RA , Sheffield SW, Butera IM, Gifford RH, Wallace MT. Multisensory integration in Cochlear implant recipients. Ear Hear. 2017:38(5):521–538. 10.1097/AUD.0000000000000435. 28399064 PMC5570631

[ref74] Tees RC . Effects of early auditory restriction in the rat on adult pattern discrimination. Journal of Comparative and Physiological Psychology. 1967:63(3):389–393. 10.1037/h0024619. 6064381

[ref75] Triplett JW , Phan A, Yamada J, Feldheim DA. Alignment of multimodal sensory input in the superior colliculus through a gradient-matching mechanism. J Neurosci. 2012:32(15):5264–5271. 10.1523/JNEUROSCI.0240-12.2012. 22496572 PMC3342701

[ref76] Wallace MT , Stein BE. Development of multisensory neurons and multisensory integration in cat superior colliculus. J Neurosci. 1997:17(7):2429–2444. 10.1523/JNEUROSCI.17-07-02429.1997. 9065504 PMC6573512

[ref77] Wallace MT , Stein BE. Onset of cross-modal synthesis in the neonatal superior colliculus is gated by the development of cortical influences. J Neurophysiol. 2000:83(6):3578–3582. 10.1152/jn.2000.83.6.3578. 10848574

[ref78] Wallace MT , Stein BE. Sensory and multisensory responses in the newborn monkey superior colliculus. J Neurosci. 2001:21(22):8886–8894. 10.1523/JNEUROSCI.21-22-08886.2001. 11698600 PMC6762279

[ref79] Wallace MT , Stein BE. Early experience determines how the senses will interact. J Neurophysiol. 2007:97(1):921–926. 10.1152/jn.00497.2006. 16914616

[ref80] Wallace MT , Meredith MA, Stein BE. Converging influences from visual, auditory, and somatosensory cortices onto output neurons of the superior colliculus. J Neurophysiol. 1993:69(6):1797–1809. 10.1152/jn.1993.69.6.1797. 8350124

[ref81] Wallace MT , McHaffie JG, Stein BE. Visual response properties and visuotopic representation in the newborn monkey superior colliculus. J Neurophysiol. 1997:78(5):2732–2741. 10.1152/jn.1997.78.5.2732. 9356422

[ref82] Wallace MT , Meredith MA, Stein BE. Multisensory integration in the superior colliculus of the alert cat. J Neurophysiol. 1998:80(2):1006–1010. 10.1152/jn.1998.80.2.1006. 9705489

[ref83] Wallace MT , Perrault TJ Jr, Hairston WD, Stein BE. Visual experience is necessary for the development of multisensory integration. J Neurosci. 2004a:24(43):9580–9584. 10.1523/JNEUROSCI.2535-04.2004. 15509745 PMC6730167

[ref84] Wallace MT , Roberson GE, Hairston WD, Stein BE, Vaughan JW, Schirillo JA. Unifying multisensory signals across time and space. Exp Brain Res. 2004b:158(2):252–258. 10.1007/s00221-004-1899-9. 15112119

[ref85] Wallace MT , Carriere BN, Perrault TJ, Vaughan JW, Stein BE. The development of cortical multisensory integration. J Neurosci. 2006:26(46):11844–11849. 10.1523/JNEUROSCI.3295-06.2006. 17108157 PMC6674880

[ref86] Wang Z , Yu L, Xu J, Stein BE, Rowland BA. Experience creates the multisensory transform in the superior colliculus. Front Integr Neurosci. 2020:14:18. 10.3389/fnint.2020.00018. 32425761 PMC7212431

[ref87] Withington-Wray DJ , Binns KE, Dhanjal SS, Brickley SG, Keating MJ. The maturation of the superior Collicular map of auditory space in the Guinea pig is disrupted by developmental auditory deprivation. Eur J Neurosci. 1990:2(8):693–703. 10.1111/j.1460-9568.1990.tb00459.x. 12106287

[ref88] Xu J , Yu L, Rowland BA, Stanford TR, Stein BE. Incorporating cross-modal statistics in the development and maintenance of multisensory integration. J Neurosci. 2012:32(7):2287–2298. 10.1523/JNEUROSCI.4304-11.2012. 22396404 PMC3561931

[ref89] Xu J , Yu L, Rowland BA, Stanford TR, Stein BE. Noise-rearing disrupts the maturation of multisensory integration. Eur J Neurosci. 2014:39(4):602–613. 10.1111/ejn.12423. 24251451 PMC3944832

[ref90] Xu J , Yu L, Stanford TR, Rowland BA, Stein BE. What does a neuron learn from multisensory experience? J Neurophysiol. 2015:113(3):883–889. 10.1152/jn.00284.2014. 25392160 PMC4312864

[ref91] Xu J , Yu L, Rowland BA, Stein BE. The normal environment delays the development of multisensory integration. Sci Rep. 2017:7(1):4772. 10.1038/s41598-017-05118-1. 28684852 PMC5500544

[ref92] Yu L , Rowland BA, Stein BE. Initiating the development of multisensory integration by manipulating sensory experience. J Neurosci. 2010:30(14):4904–4913. 10.1523/JNEUROSCI.5575-09.2010. 20371810 PMC2858413

[ref93] Yu L , Xu J, Rowland BA, Stein BE. Development of cortical influences on superior colliculus multisensory neurons: effects of dark-rearing. Eur J Neurosci. 2013:37(10):1594–1601. 10.1111/ejn.12182. 23534923 PMC3660411

[ref94] Yu L , Cuppini C, Xu J, Rowland BA, Stein BE. Cross-modal competition: the default computation for multisensory processing. J Neurosci. 2019:39(8):1374–1385. 10.1523/JNEUROSCI.1806-18.2018. 30573648 PMC6381255

