# Supplementary material for: The brain can develop conflicting multisensory principles to guide behavior
Source: Cereb Cortex. 2024 Jun 15;34(6):bhae247. doi: 10.1093/cercor/bhae247 (PMC11179994; doi:10.1093/cercor/bhae247)
Supplement: Supplementary_Tables_bhae247 [file supplementary_tables_bhae247.pdf]

	**Supplementary Table 1:** Experiment 1: Performance (Left Hemifield Only) (Congruent A-45°/V-45° exposure position highlighted)								
	Location	-60°		-45°		-30°		-15°	
	Condition	Pre	Post	Pre	Post	Pre	Post	Pre	Post
A	Performance	38±4.4	36±2	36±5.6	37±1.3	36±2.8	35±1.8	28±2.6	31±0.7
	Detection	47±4.3	58±11	50±3.9	65±6.6	55±2.2	71±12	44±4.2	57±2.4
	Localization	79±2	66±10	71±7.2	58±3.8	65±5.4	52±7.4	64±1.2	55±3.3
V	Performance	30±1.8	31±2.4	31±3	33±1.8	34±1.7	32±1.2	35±1.6	35±1.3
	Detection	35±2.1	41±3.5	37±2	49±2.4	44±5.5	47±1.8	52±2.1	50±2.3
	Localization	85±5.1	74±2.9	84±8.1	69±5	81±7.5	68±5	67±0.8	69±1.5
SF	Performance	56±3.9	56±1.7	56±5.4	60±1.8	58±2.9	57±1.8	53±1.8	56±1.6
	Detection	66±3.9	75±7.6	69±1.6	82±3.7	75±3.3	85±5.6	73±2.3	79±0.4
	Localization	86±2.1	76±5.1	81±6.4	73±1.4	78±1.3	67±2.7	73±0.2	71±2.3
VA	Performance	50±4.7	77±8.7	46±8.2	89±1.8	38±5.1	80±5.3	36±7.3	57±1.8
	Detection	59±5	85±9.8	60±6.9	95±2.9	59±2.1	94±1.2	58±8.5	83±4.1
	Localization	85±2.5	91±3	75±5.9	93±2.4	64±6.2	85±5.1	62±8.4	70±4.6
ME_{SF}	Performance	-12±1.3	38±7.4	-20±5.1	49±0.9	-35±3.6	41.2±3	-33±7.1	2.4±3.1
	Detection	-11±2.8	15±8.1	-14±5.1	16±2.1	-21±1.7	11±4.1	-21±6.4	4.9±2.8
	Localization	-0.6±2.9	21±7.1	-7.1±2.4	28±1.6	-18±4.2	28±7	-15±6.8	-2.1±3.4
Z-score (p)	Performance	-1.9 (0.0336)	6.3 (<0.001)	-2.9 (0.0015)	8.6 (<0.001)	-5.1 (<0.001)	6.5 (<0.001)	-4 (0.0001)	0.2 (0.6064)
	Detection	-2.1 (0.0168)	4.7 (<0.001)	-3.3 (0.0008)	5.5 (<0.001)	-5 (<0.001)	3.8 (<0.001)	-4.6 (<0.001)	1.3 (0.9259)
	Localization	-0.3 (0.3693)	3.7 (<0.001)	-0.8 (0.2216)	5.5 (<0.001)	-3 (0.0022)	4.8 (<0.001)	-1.8 (0.039)	-0.7 (0.2265)

	**Supplementary Table 2:** Experiment 1: Performance (Right Hemifield Only) (Modality-Specific A-45°, V-45° exposure position highlighted)								
	Location	15°		30°		45°		60°	
	Condition	Pre	Post	Pre	Post	Pre	Post	Pre	Post
A	Performance	29±5.4	31±0.7	38±2.2	31±2.9	40±6.2	32±5.3	39±0.9	33±2.4
	Detection	57±7.4	59±8.2	61±3.7	47±5.7	60±6.2	45±5.7	56±2.4	45±7.5
	Localization	52±13.1	55±7	63±3.7	67±3.9	66±7.2	70±5	69±1.7	76±8.8
V	Performance	34±4.1	35±3.5	36±8	33±1.3	41±5.5	32±5	42±4	31±2.4
	Detection	43±6.4	48±6.4	48±7.7	41±5.5	49±5.4	42±6.4	53±3.2	41±7.5
	Localization	81±2.4	73±5.6	73±5.5	81±7.8	84±2	76±3.3	80±5.5	77±8.6
SF	Performance	53±6.2	56±2.1	61±6	54±2.2	64±6.8	54±6.8	65±2.1	53±3.5
	Detection	76±3.9	79±3.7	79±5.1	69±4.8	79±4.9	68±6.6	79±2.2	67±8
	Localization	70±7.5	71±5.3	77±3	79±3.1	80±5	79±3.4	82±2.6	81±5.5
VA	Performance	41±7.4	48±3.1	47±8	49±2.4	54±9.3	52±3.5	60±3	44±5.3
	Detection	65±4.7	75±9.8	64±5.2	69±3.5	66±7.4	69±10.1	71±4.4	59±8.8
	Localization	62±7.9	65±6.8	72±6.8	70±4	81±5.1	77±7.5	84±1.5	75±2.1
ME_{SF}	Performance	-24±2.9	-14±3.4	-24±4.3	-9.7±2.5	-16±3.6	-1.3±3.6	-8±2.1	-18±3.3
	Detection	-15±2.3	-5.8±5.1	-19±1.6	0.8±1.7	-16±4.9	1.4±3.3	-10±1.9	-11±5
	Localization	-11±1.1	-8.2±1.6	-6.6±3.8	-11±1.1	0.7±2.9	-1.5±6.9	2.8±1	-7±3.2
Z-score (p)	Performance	-3.9 (<0.001)	-2.1 (0.0249)	-3.9 (<0.001)	-1.4 (0.0912)	-2.1 (0.0205)	-0.9 (0.1954)	-1.5 (0.0649)	-2.6 (0.0058)
	Detection	-4 (<0.001)	-1.5 (0.0884)	-4.5 (<0.001)	0.4 (0.6956)	-3.5 (0.0006)	0 (0.5044)	-2.9 (0.0025)	-2.2 (0.0196)
	Localization	-2.3 (0.0122)	-1.6 (0.0602)	-1.5 (0.0682)	-2.4 (0.0098)	0.4 (0.6689)	-1.3 (0.1008)	0.8 (0.7859)	-1.6 (0.0611)

	**Supplementary Table 3:** Experiment 1: Response Bias (congruent A-45°/V-45° and modality specific A45°, V45° exposure positions highlighted)								
	Location	-60°	-45°	-30°	-15°	15°	30°	45°	60°
A	Pre	8.7±5.4	5.8±5.7	-3.4±0.9	0.5±7.7	3.4±1	2.3±4.3	-10±4.2	-12±4.4
	Post	8.8±3.2	1.2±1.6	-0.1±5.1	-2.5±3.5	5±3.9	-6.8±4.3	-7.5±2.9	-7.5±5.1
	Cohen's d	0.02	-0.78	0.64	-0.35	0.4	-1.5	0.49	0.67
V	Pre	5.9±0.8	9.3±8.1	2.4±1	3.9±1.6	-0.6±3.6	-8.1±2.4	-5.1±3.7	-7.5±2.3
	Post	4.2±4.2	5.4±1.7	1.8±2.8	-0.9±1.5	-0.6±1.6	-2.0±1.3	-5.7±3.9	-5.4±0.6
	Cohen's d	-0.4	-0.47	-0.2	-2.19	0	2.23	-0.11	0.88
SF	Pre	5.8±2.6	5.7±5.5	-0.9±0.4	1.4±3.2	1.4±1.5	-1.3±2.5	-5.6±2.9	-6.4±1.2
	Post	4.7±2	1.5±0.9	-0.6±2.6	-1.3±1.6	2.6±1.9	-3.7±2.5	-5.1±2.2	-5.3±2.4
	Cohen's d	-0.34	-0.75	0.11	-0.75	0.5	-0.68	0.14	0.41
VA	Pre	3.1±2.4	5.2±5.3	0.6±6.4	-7.9±4	2.9±2.9	-0.1±0.9	-3.8±4.3	-3.1±1.3
	Post	1.9±0.8	0.9±0.6	-1.1±0.6	-2.4±2.2	-1.3±0.8	-3.9±2.3	-2.7±0.7	-6.3±1.9
	Cohen's d	-0.47	-0.81	-0.26	1.2	-1.4	-1.54	0.25	-1.39

	**Supplementary Table 4:** Experiment 2: Performance (Left Hemifield Only) (Congruent A-45°/V-45° exposure position highlighted)						
	Location	-45°		-30°		-15°	
	Condition	Pre	Post	Pre	Post	Pre	Post
A	Performance	39±2.7	39±2.4	38±2.3	34±2.3	37±6.4	39±1.8
	Detection	81±2.4	78±3.5	79±3.7	68±7	71±10.1	63±3.7
	Localization	48±3.1	50±3.5	48±5.3	50±2.5	51±2.5	62±1.8
V	Performance	44±2.3	22±1.2	47±8.2	29±2.9	39±2.7	38±4.2
	Detection	67±8.4	39±3.5	74±2	65±5.7	67±1.3	64±9
	Localization	68±4.6	57±3.2	63±6.9	46±5.2	58±3.3	60±4
SF	Performance	68±3.4	53±1.9	71±4.6	56±1.4	61±5.2	62±3.9
	Detection	93±2.6	87±2.6	95±0.7	88±3.9	91±3.5	86±4.3
	Localization	72±2.7	61±2.5	75±5.4	64±3.3	68±3.6	73±2.7
VA	Performance	56±5	84±3.5	45±2.4	74±3.1	43±4.7	49±6.6
	Detection	81±3.7	98±2	83±4.7	91±1.8	78±5.3	78±4.2
	Localization	69±5.9	86±2.2	56±6	81±2.2	55±2.9	62±5.3
ME_{SF}	Performance	-17±2.8	59±4.3	-36±2	32±2.4	-30±3.5	-23±4.3
	Detection	-13±1.1	13±0.9	-13±3.1	4.1±2.2	-14±1.9	-9.1±2.6
	Localization	-5.1±3.6	41±4.4	-27±2.4	27±4.6	-18±3.9	-15±3.9
Z-score (p)	Performance	-3 (0.0024)	7.6 (<0.001)	-6.9 (<0.001)	4.3 (<0.001)	-4.7 (<0.001)	-3.4 (0.0005)
	Detection	-6.2 (<0.001)	4.1 (<0.001)	-6.4 (<0.001)	1 (0.8793)	-5.3 (<0.001)	-3 (0.0021)
	Localization	-0.9 (0.194)	5.7 (<0.001)	-5.6 (<0.001)	4.2 (<0.001)	-3.2 (0.0009)	-2.4 (0.0088)

	**Supplementary Table 5:** Experiment 2: Performance (Right Hemifield Only)						
	Location	15°		30°		45°	
	Condition	Pre	Post	Pre	Post	Pre	Post
A	Performance	42±5.8	35±4.1	41±1.3	41±3.3	37±1.8	42±2
	Detection	79±5.8	68±4.2	78±2.3	65±3.7	73±1.3	75±1.3
	Localization	53±6	52±5.8	52±2.4	62±1.8	50±1.8	56±2.3
V	Performance	56±5.8	32±4.2	53±0.7	32±9.9	49±3.5	17±5.7
	Detection	81±3.5	61±3.5	77±2.9	48±11	69±5.7	32±3.1
	Localization	69.1±3	52±3.2	70±2.4	63±5.1	71±6	51±10
SF	Performance	74±5.9	56±3.5	76±0.6	61±8.2	68±2.4	52±4.9
	Detection	96±1.4	87±2.7	95±1	81±5.8	92±1.4	83±1.7
	Localization	77±5.2	64±2.3	80±1.2	74±5.1	74±3	62±5.3
VA	Performance	49±8.5	76±1.2	53±2.4	61±8.4	45±5.9	48±5.3
	Detection	75±7.7	93±0.7	81±1.3	85±6.4	70±7.2	73±6.7
	Localization	65±4.4	81±1.4	66±3.8	71±5	65±4.2	66±2.7
ME_{SF}	Performance	-34±4.6	36±7.6	-30±2.3	0.1±1.5	-33±7.6	-7.2±2.1
	Detection	-22±4.9	7.1±2.3	-14±0.9	4.1±1.2	-23±6.3	-12±5
	Localization	-16±2.4	27±4.4	-18±3	-3.9±0.4	-12±6.7	6.8±5
Z-score (p)	Performance	-7.1 (<0.001)	4.8 (<0.001)	-6.5 (<0.001)	-0.1 (0.4811)	-5.9 (<0.001)	-1 (0.1864)
	Detection	-12.8 (<0.001)	2.1 (0.0073)	-7.5 (<0.001)	0.9 (0.834)	-9.6 (<0.001)	-3.3 (0.0012)
	Localization	-3.6 (0.0004)	4.1 (<0.001)	-4.4 (<0.001)	-0.8 (0.2234)	-2.5 (0.0084)	0.8 (0.7747)

	**Supplementary Table 6:** Experiment 2: Spatially Disparate Performance (Left Hemifield Only)								
	A Location	-45°		-15°		-15°		-15°	
	V Location	-15°		-60°		-45°		-30°	
	Condition	Pre	Post	Pre	Post	Pre	Post	Pre	Post
SF	Performance	41±3.8	45±0.8	N/A	55±5.8	45±5.6	42±3.8	N/A	60±6.1
	Detection	94±1	92±2.4	N/A	86±7.1	92±2.1	77±3.5	N/A	97±3.0
	Localization	44±4	48±1.6	N/A	64±11	48±5.2	54±3	N/A	62±9.9
VA	Performance	38±4.2	35±1.3	N/A	46±4.3	27±3.5	28±5.8	N/A	45±6.2
	Detection	83±1.8	88±4.2	N/A	92±4.3	84±3.1	88±7.2	N/A	92±4.2
	Localization	46±4.7	40±3.2	N/A	50±2.5	33±3.8	32±6.3	N/A	49±4.8
ME_{SF}	Performance	-7.3±5.8	-22±1.7	N/A	-17±0.6	-38.3±2.4	-35±5.9	N/A	-25±2.4
	Detection	-11.7±1.2	-4.5±2.5	N/A	4.8±2.2	-8.1±3.3	14±4.2	N/A	-5.6±2.8
	Localization	5±6.7	-18±3	N/A	-18±2.0	-33±0.5	-42±6.3	N/A	-21±2.8
Z-score (p)	Performance	-0.8 (0.2311)	-2.4 (0.0103)	N/A	-0.9 (0.2319)	-4.2 (<0.001)	-3.5 (0.0002)	N/A	-1.5 (0.0501)
	Detection	-5.5 (<0.001)	-1.9 (0.0489)	N/A	0.80 (0.1281)	-2.7 (0.0112)	3.1 (<0.001)	N/A	-1.3 (0.2015)
	Localization	0.5 (0.6722)	-2.1 (0.021)	N/A	-1.4 (0.0706)	-3.9 (0.0001)	-4.9 (<0.001)	N/A	-1.3 (0.0918)

	**Supplementary Table 7:** Experiment 2: Spatially Disparate Performance (Right Hemifield Only) (Disparate A15°/V45° Exposure position highlighted)								
	A Location	15°		15°		15°		45°	
	V Location	30°		45°		60°		15°	
	Condition	Pre	Post	Pre	Post	Pre	Post	Pre	Post
SF	Performance	N/A	51±6.6	49±4.8	39±3.6	N/A	51±5.8	40±0.4	44±2.2
	Detection	N/A	89±6.4	94±0.8	78±3.3	N/A	85±7.3	95±1.3	90±1.1
	Localization	N/A	58±11	52±4.8	50±5.4	N/A	61±11	42±0.2	48±1.8
VA	Performance	N/A	83±2.1	49±5.2	67±3.5	N/A	58±4.3	41±5.7	33±6.8
	Detection	N/A	97±0.69	89±1.3	90±1.2	N/A	88±3.0	81±0.7	86±2
	Localization	N/A	86±1.9	56±5.9	75±4.9	N/A	66±6.0	51±7.2	38±7.3
ME_{SF}	Performance	N/A	67±10	-0.1±1.2	75±4.8	N/A	14±3	4.7±10.4	-26±8.1
	Detection	N/A	7.1±1.5	-6±0.6	16±3.5	N/A	2.5±2.4	-14±0.3	-4.6±1.2
	Localization	N/A	65±20	6.3±1.9	52±5.3	N/A	20±11	21.8±12.2	-24±8.3
Z-score (p)	Performance	N/A	3.1 (0.0003)	0 (0.5455)	7.2 (<0.001)	N/A	0.7 (0.1998)	0.5 (0.7125)	-2.7 (0.0039)
	Detection	N/A	1.3 (0.0657)	-2.5 (0.0144)	3.5 (<0.001)	N/A	0.45 (0.2775)	-7.3 (<0.001)	-1.7 (0.0622)
	Localization	N/A	2.6 (0.0039)	0.7 (0.7705)	5.5 (<0.001)	N/A	0.51 (0.3275)	2.2 (0.989)	-2.4 (0.0075)

	**Supplementary Table 8:** Experiment 2: Response Bias (Spatially Congruent Only) (exposure position highlighted)						
	Location	-45°	-30°	-15°	15°	30°	45°
A	Pre	7±2.7	-1±1.8	-2.9±1.4	2.8±1	-1.8±1.7	-9.6±0.3
	Post	3.5±1.5	-2±0.9	-3.5±0.7	1.9±1.2	2±0.1	-7.5±0.8
	Cohen's d	-1.13	-0.5	-0.38	-0.58	2.23	2.46
V	Pre	5.5±1.3	2.4±0.9	1±1.3	1.8±2	-0.6±0.8	-5.2±1.1
	Post	16±1.9	0.6±1.7	-2.8±0.7	-2.1±3.3	-4.6±3.5	-20±8.2
	Cohen's d	4.56	-0.94	-2.57	-1.01	-1.11	-1.79
SF	Pre	3.7±1	-0.1±0.9	-1.6±0.6	2±1	-0.6±0.8	-4.6±0.2
	Post	3.9±1.2	-1.6±0.7	-2.6±0.5	1±1.3	0±1	-7.5±1.7
	Cohen's d	0.13	-1.32	-1.28	-0.61	0.47	-1.69
VA	Pre	5.4±1.8	1.4±2.4	-0.9±2	1.2±1.3	-0.7±1.6	-6.8±1.4
	Post	3.5±0.5	-1.4±0.9	-2.8±1.4	-0.8±0.8	-0.8±1.5	-6.2±0.5
	Cohen's d	-1.02	-1.09	-0.78	-0.64	-0.05	0.4

	**Supplementary Table 9:** Experiment 2: Response Bias (Spatially Disparate Only) (Positive Values indicate bias towards visual stimulus location) (exposure position highlighted)								
	A Location	-45°	-15°	-15°	-15°	15°	15°	15°	45°
	V Location	-15°	-60°	-45°	-30°	30°	45°	60°	15°
SF	Pre	9.9±2.2	N/A	9.0±2.3	N/A	N/A	8.0±1.5	N/A	13±0.69
	Post	7.1±1.0	0.09±1.8	5.8±0.3	4.8±0.49	3.6±1.0	3.5±2.0	0.72±1.5	11±1.3
	Cohen's d	-1.16	N/A	-1.38	N/A	N/A	-1.8	N/A	-1.36
VA	Pre	13±2.2	N/A	10±2.3	N/A	N/A	6.6±1.5	N/A	11±2.3
	Post	11±1.6	4.7±1.7	8.5±1.6	4.7±0.66	0.1±0.21	0.75±1.2	2.2±1.4	14±2.7
	Cohen's d	-0.74	N/A	-0.54	N/A	N/A	-3.05	N/A	0.85
